# Effect of *Chlorella*-derived multicomponent supplementation on maximal oxygen uptake and serum vitamin B_2_ concentration in young men

**DOI:** 10.3164/jcbn.17-36

**Published:** 2017-08-18

**Authors:** Asako Zempo-Miyaki, Seiji Maeda, Takeshi Otsuki

**Affiliations:** 1 Faculty of Sport and Health Sciences, Ryutsu Keizai University, 120 Ryugasaki, Ibaraki 301-8555, Japan; 2 Faculty of Health and Sport Sciences, University of Tsukuba, 1-1-1 Tennoudai, Tsukuba, Ibaraki 305-8577, Japan

**Keywords:** aerobic capacity, *Chlorella*, daily food intake frequency, maximal oxygen uptake, vitamin B_2_

## Abstract

*Chlorella* is a unicellular green alga that contains high levels of proteins, vitamins and minerals. The present study investigated the effects of a 4-week *Chlorella*-derived multicomponent supplementation on maximal oxygen uptake and circulating vitamin B_2_ levels in healthy men. Thirty-four participants were randomly divided into two groups: placebo or *Chlorella*. Prior to the intervention, we observed that the intake of several minerals and soluble vitamins did not satisfy the nutrient requirements of either group by assessing the frequency of daily food intake. There was a significant negative relationship between the pre-intervention maximal oxygen uptake and serum vitamin B_2_ concentrations in all subjects (*r* = −0.372). Maximal oxygen uptake significantly increased after *Chlorella* supplementation (before vs after, 42.1 ± 1.5 vs 44.9 ± 1.6 ml/kg/min), while serum vitamin B_2_ concentrations did not (14.6 ± 0.9 vs 14.0 ± 0.9 µg/L). In conclusion, *Chlorella*-derived multicomponent supplementation increases maximal oxygen uptake in individuals with an insufficient micronutrient status, although there was no association between the increase in aerobic capacity and serum levels of vitamin B_2_.

## Introduction

A well-balanced diet is one of the most important factors for being fit and leading a healthy life. In the last few decades, dietary social problems, including skipping breakfast, eating meals irregularly and consuming fast food, have become increasingly common and have led to malnutrition in young individuals.^(1–3)^ In particular, university students living away from home are at a high risk of poor nutrition.^(4)^ The National Health and Nutrition Examination Survey by the Ministry of Health, Labour and Welfare of Japan reported that many young Japanese men have poor dietary habits, including skipping breakfast or having a low vegetable intake, as compared with women and/or other generations. In particular, the average vegetable intake among Japanese men aged 20–29 years in 2014 was 237 g/day, despite the recommended intake being 350 g/day; only 19% of young Japanese men achieved the recommended level. Fujii *et al.*^(5)^ reported that Japanese college students who regularly skip breakfast also tend to have a poor vegetable intake. Such dietary problems are not unique to Japan, but also occur throughout the world.^(1,2,6)^ As a solution to such dietary problems in young individuals, dietary supplements may be effective at overcoming nutrient deficiencies by providing a better nutrient balance.

As a result of an evidence-based analysis, the Academy of Nutrition and Dietetics, Dietitians of Canada and American College of Sports Medicine have stated that well-balanced nutrition is necessary for maximal exercise performance. Exercise training increases micronutrient requirements, and a daily multivitamin and multimineral supplement would benefit athletes who have a poor micronutrient status.^(7)^ In our previous cross-over study, a *Chlorella*-derived multicomponent supplementation increased the aerobic exercise capacity of healthy young individuals.^(8)^ However, our previous study did not explore the nutritional status of the participants or the mechanisms underlying the increase in aerobic exercise capacity.

*Chlorella* is a unicellular green alga that contains high levels of proteins, chlorophylls, vitamins, minerals and dietary fiber. A lack of vitamin B_1_, B_2_, B_6_ and C for 10 weeks decreased the peak oxygen uptake in young individuals.^(9)^ In addition, vitamin B_2_ intake is an important factor regarding endurance exercise capacity.^(10)^ Therefore, we hypothesized that *Chlorella*-derived supplementation would increase maximal oxygen uptake (V̇O_2_max) by improving the levels of vitamin B_2_ and other micronutrients in individuals with an insufficient micronutrient status. To test this hypothesis, we assessed daily food intake frequency and examined the effects of a 4-week *Chlorella*-derived supplementation on the V̇O_2_max and circulating vitamin B_2_ levels in healthy young men using a double-blinded study design.

## Materials and Methods

### Subjects and experimental design

A total of 34 young men volunteered to participate in this study. After pre-supplementation tests, the subjects were randomly divided into the placebo (*n* = 17) and *Chlorella* (*n* = 17) groups in a double-blinded manner. During the trial, the participants self-administered 30 tablets of either *Chlorella* or the placebo per day (15 tablets twice daily, after breakfast and dinner) for 4 weeks. This dosage was in accordance with our previous studies^(8,11,12)^ and the general recommended dosage for Japanese consumers. The participants were asked not to modify their lifestyle during the trial period. Post-supplementation measurements and blood sampling were performed one day after the final tablet intake.

This study was approved by the Ethics Committee of Ryutsu Keizai University. The protocol of this study conformed to the principles of the Declaration of Helsinki. All participants provided written informed consent prior to study initiation.

A power calculation was performed for Pearson’s correlation coefficients in all participants and for repeated measures two-way analysis of variance (ANOVA) using G*Power 3.^(13)^ The sample size of this study was enough to detect the correlation and effects of supplementation at 90% power and with an α of 5% when effect size was assumed as medium.^(14)^

The *Chlorella* (SunChlorella A; Sun Chlorella Corp., Kyoto, Japan) and placebo tablets used in this study were the same as those used in our previous studies.^(8,11,12)^ Briefly, the *Chlorella* tablet comprised dried *Chlorella pyrenoidosa* powder as the main ingredient. The mass of each *Chlorella* tablet was 200 mg. The nutritional values per 100 g of the tablet were as follows: energy, 399 kcal; water, 5.3 g; protein, 60.8 g; lipid, 9.2 g; saccharide, 6.3 g; dietary fiber, 11.9 g; and ash, 6.5 g (including 5.61 mg of vitamin B_2_). The main ingredient of the placebo tablet was lactose. The color and shape of the placebo tablet were similar to that of the *Chlorella* tablet, but the protein (2.0 g), dietary fiber (1.1 g) and ash (2.2 g) contents in the placebo tablet were markedly lower than those in the *Chlorella* tablet.

### Maximal exercise testing

Subjects refrained from alcohol consumption starting on the day before testing, intense physical activity and caffeine consumption starting at 9 p.m. on the day before testing, and drinking and eating excluding water for 1 h before the testing.

Maximal exercise testing involved incremental cycling to exhaustion (4 min at 70 W, with a 30 W increase every min), with monitoring of breath-by-breath oxygen uptake and carbon dioxide production (AE300S; Minato Medical Science, Osaka, Japan), heart rate (HR) calculation using 3-lead electrocardiography (LRR-03; GMS, Tokyo, Japan) and ratings of perceived exertion (RPE, Borg’s 6–20 scale). We considered a participant as having achieved maximal exertion when at least two of the following four criteria were met: 1) a plateau in oxygen uptake with increasing exercise intensity (<100 ml/min); 2) achievement of age-predicted maximal HR (±10 bpm); 3) a respiratory exchange ratio of at least 1.15; and 4) an RPE of at least 18 units.^(8)^ In our laboratory, the day-to-day coefficient of variation for V̇O_2_max was 4.7 ± 4.0%.

### Serum vitamin B_2_ analysis

Serum vitamin B_2_ concentration was measured using a microbiological test. The subjects refrained from alcohol consumption from the day before testing. Blood samples were collected from the antecubital vein by venipuncture after an overnight fast, placed in chilled tubes and centrifuged at 3,000 rpm for 15 min at 4°C. The plasma was stored at −80°C prior to analysis. Serum concentrations of vitamin B_2_ were determined using a commercial kit including a microtiter plate coated with *Lactobacillus rhamnosus*, according to the manufacturer’s protocol (ID-Vit^®^ Vitamin B_2_; Immundiagnostik AG, Bensheim, Germany).

### Dietary intake

Dietary habits were assessed using a validated, brief, self-administered dietary history questionnaire (BDHQ).^(15)^ The BDHQ is a four-page structured questionnaire that enquires about the consumption frequency of 58 food and beverage items, with specified serving sizes described in terms of the natural portion or the standard weight and volume measurement of servings commonly consumed in the general Japanese population. Validation of the BDHQ was performed using 16-day weighed dietary records as the gold standard.^(15)^

With regard to protein, calcium, magnesium, iron, zinc, copper, retinol, vitamin B_1_, vitamin B_2_, niacin, vitamin B_6_, vitamin B_12_ and vitamin C, the intake amounts are expressed as the ratio to the recommended dietary amount (RDA) of the Dietary Reference Intake for Japanese 2015. Sodium intake is expressed as the ratio to the estimated average requirement (EAR), as the RDA of sodium was not established in the Dietary Reference Intake. For nutrients without a RDA or an EAR (n-6 fatty acid, n-3 fatty acid, potassium, phosphorus, manganese, vitamin D, vitamin K, α-tocopherol and pantothenic acid), the sufficiency rates were estimated based on the adequate intake (AI). The RDA and EAR indicate the amount that would meet the requirement for 97–98 and 50% of the population, respectively. The AI was developed when EAR and RDA could not be set because of insufficient scientific evidence and indicates the amount of nutrients required to maintain a healthy status.

### Body mass and fat mass

The subjects refrained from alcohol consumption from the day before testing. Body mass was measured and body fat mass was assessed by bioelectrical impedance analysis after an overnight fast (InBody 430; InBody Japan, Tokyo, Japan).

### Statistical analysis

Results are presented as the mean ± SE. Inter-group comparisons for patient variables before supplementation were performed using unpaired *t* tests. Relationships between two variables were assessed using Pearson’s correlation coefficient. To compare the effects of *Chlorella* tablet intake with placebo supplementation, repeated-measures two-way ANOVA was used. If a significant *F* value was found, a post-hoc Fisher’s exact test was conducted to identify the effects of placebo and *Chlorella* tablet intake. *P* values <0.05 were considered statistically significant.

## Results

Table 1 shows the anthropometric characteristics of the participants. There were no significant differences in the pre-measurement data between the *Chlorella* and placebo groups. Moreover, the height, body mass and body fat mass of both groups did not change significantly after the 4-week intervention.

Table 2 shows dietary habits of the two groups before the intervention and the nutrient intakes including *Chlorella* supplementation. There were no differences in the daily nutrient intake between the groups before the tablet intake period. Several nutrients did not satisfy the nutrient demand (i.e., RDAs, EARs or AIs) in both groups, as follows: potassium, calcium, magnesium, zinc, manganese, vitamin A, vitamin B_1_, vitamin B_2_, vitamin B_6_ and vitamin C. On the other hand, sodium was above the nutrient demand in both groups. The energy intake was lower than the demand in both groups, but the intake of protein and fat was higher than the demand. The sufficiency rate of iron, vitamin B_2_, D and K, and niacin increased by ≥20% with *Chlorella* supplementation.

**Fig. 1:**
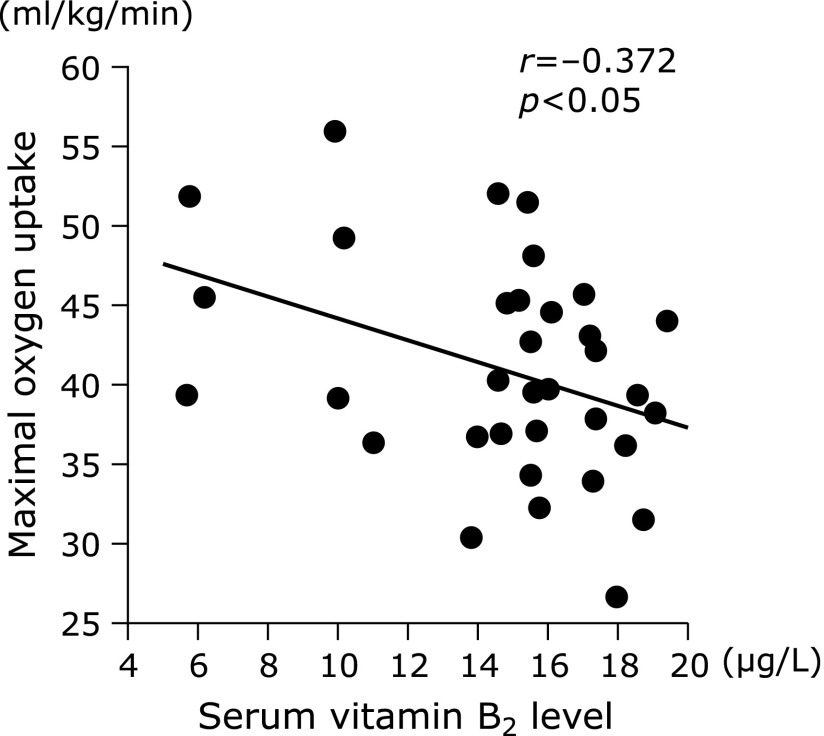
Relationship between maximal oxygen uptake and serum vitamin B_2_ concentrations in all participants before the intervention. There was a negative relationship between the maximal oxygen uptake and serum vitamin B_2_ concentrations in all participants before the intervention.

**Fig. 2:**
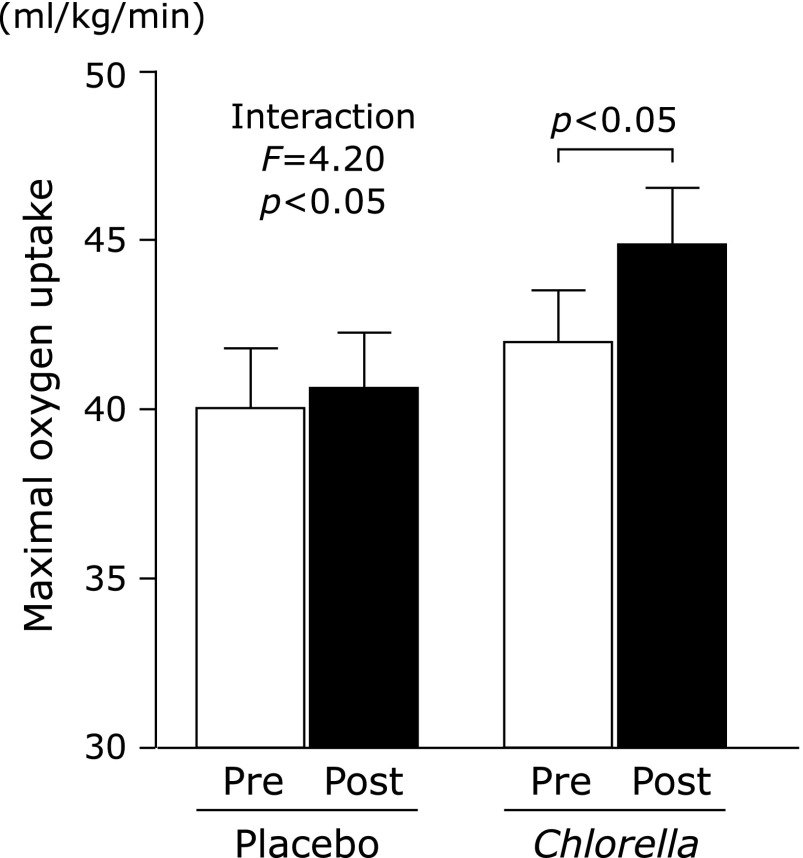
Maximal oxygen uptake pre and post intervention of the placebo and *Chlorella* groups. Values are mean ± SE. The maximal oxygen uptake was increased following *Chlorella*-derived multicomponent supplementation.

**Fig. 3:**
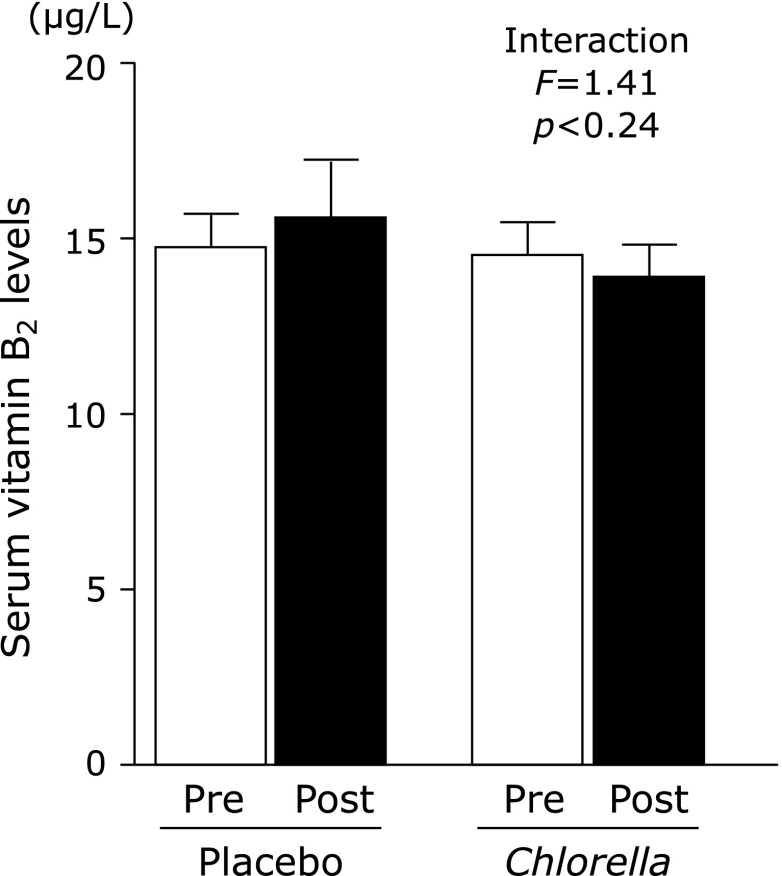
Serum vitamin B_2 _concentrations pre and post intervention of the placebo and *Chlorella* groups. Values are mean ± SE.

There was a significant negative relationship between the V̇O_2_max and serum vitamin B_2_ concentration in all participants before the intervention (Fig. 1). The V̇O_2_max prior to and following the intervention is shown in Fig. 2. There was a significant interaction in the changes between the *Chlorella* and control groups. The V̇O_2_max significantly increased following *Chlorella*-derived supplementation. Changes in vitamin B_2_ concentration before and after the intervention are shown in Fig. 3; there was no significant interaction between the *Chlorella* and control groups. Additionally, the relationship between the change in the V̇O_2_max and that of the serum vitamin B_2_ levels did not reach statistical significance in the *Chlorella* group (*r* = −0.311, *p* = 0.22).

## Discussion

We investigated the effects of a 4-week *Chlorella*-derived multicomponent supplementation on the V̇O_2_max and circulating vitamin B_2_ levels in healthy young men. Prior to the intervention, the daily intake of calcium, magnesium, vitamin B_1_, vitamin B_2_, vitamin B_6_ and vitamin C did not satisfy nutrient demands in both groups. However, vitamin B_2_ intake was markedly increased in the *Chlorella* group following supplementation. There was a significant negative relationship between the pre-intervention V̇O_2_max value and the serum levels of vitamin B_2_ in all subjects. In addition, in the *Chlorella* group, the V̇O_2_max was significantly increased following the supplementation, and serum vitamin B_2_ levels showed a decreasing trend. Therefore, we concluded that *Chlorella*-derived supplementation increases the V̇O_2_max in individuals with an insufficient micronutrient status, although there was no association between the increased V̇O_2_max and blood levels of vitamin B_2_.

In a previous crossover study, we demonstrated that *Chlorella*-derived supplementation increased the V̇O_2_max in young men (*n* = 7) and women (*n* = 3).^(8)^ In this study, we investigated the effects of a 4-week *Chlorella*-derived supplementation on aerobic exercise capacity in 34 young men, thus increasing the number of participants and eliminating any confounding effects associated with the female menstrual cycle. In addition, the current study assessed the daily food intake frequency in order to clarify the participants’ dietary habits. This study supports the results of the previous study,^(8)^ as a 4-week *Chlorella*-derived supplementation significantly increased the V̇O_2_max in young men with inadequate dietary habits.

In the present cross-sectional analysis of pre-supplementation values, we showed for the first time that there was a significant negative correlation between the V̇O_2_max and serum vitamin B_2_ concentrations. These results suggest that individuals with a higher aerobic capacity metabolize more vitamin B_2_ than do those with a lower aerobic capacity. Previously, Belko *et al.*^(16,17)^ reported that various lifestyle modifications, including dietary modification and/or regular exercise, would lead to increased vitamin B_2_ demands. Moreover, Manore^(18)^ demonstrated that considerably more vitamin B_2_ was required when dieting was combined with exercise. It is possible that the lower circulating levels of vitamin B_2_ in individuals with a higher aerobic capacity were due to the higher constitutive usage of vitamin B_2_.

Before the intervention, the sufficiency rate of vitamin B_2_ was 67% in the *Chlorella* group. However, after the *Chlorella*-derived supplementation, this rate was increased to 86%. No other nutrients demonstrated such a dramatic increase in the sufficiency rate. Therefore, this study subsequently investigated serum vitamin B_2_ concentrations. Vitamin B_2_ is necessary for the synthesis of two important coenzymes, flavin mononucleotide and flavin adenine dinucleotide. These coenzymes are especially important for the metabolism of glucose, fatty acids, glycerol and amino acids. In a previous study, reduced intake of vitamins B_1_, B_2_, B_6_ and C was associated with a decreased aerobic exercise capacity in young men.^(9)^ In addition, Peluchetti *et al.*^(10)^ demonstrated that vitamin B_2_ supplementation dramatically increased aerobic exercise capacity (before: 10.5–14.4 ml/kg/min; after: 30.5–39.3 ml/kg/min) in patients with multiple acyl-coenzyme A dehydrogenase deficiency. These results suggest that vitamin B_2_ and other soluble vitamins are important factors associated with the V̇O_2_max. However, the present study did not show a significant change in circulating vitamin B_2_ levels following *Chlorella* supplementation. Dietary modifications and/or regular exercise increase the vitamin B_2_ demand,^(16–18)^ and the circulating level of vitamin B_2_ was lower in participants with a higher aerobic capacity in the present study. Therefore, the additional vitamin B_2_ supplied by the *Chlorella*-derived tablet and the increased vitamin B_2_ demand due to an increase in aerobic capacity may have counteracted each other, resulting in no change in the vitamin B_2_ concentration.

The National Health and Nutrition Examination Survey by the Ministry of Health, Labour and Welfare of Japan has reported that vegetable intake in Japan has declined in recent years across all generations, and the decline has been particularly pronounced in men. Indeed, some vitamins and minerals (i.e., potassium, calcium, magnesium, zinc, manganese, retinol, vitamin B_1_, vitamin B_2_, vitamin B_6_ and vitamin C) were deficient in the participants of the present study. The Academy of Nutrition and Dietetics, Dietitians of Canada and American College of Sports Medicine^(7)^ have suggested that calcium, iron, zinc, magnesium, vitamin B, vitamin C and vitamin E are important nutrients for athletes. The *Chlorella*-derived supplement used in the present study contained many vitamins and minerals (e.g., vitamin A, B vitamins, vitamin C, vitamin E, calcium, folate, iron, pantothenic acid, potassium, magnesium and zinc). In particular, it should be noted that the sufficiency rate of vitamin B_2_ was essentially improved following self-administration of *Chlorella*-derived tablets.

It is unclear whether vitamin B_2_ directly affects aerobic capacity. Future investigations should focus on whether the bioavailability of vitamin B_2_ is associated with the observed increase in the V̇O_2_max following *Chlorella*-derived tablet intake. Since B vitamins are water-soluble, changes in blood and urine vitamin B_2_ concentrations should be examined.

In conclusion, we found a significant negative relationship between the V̇O_2_max and serum vitamin B_2_ concentrations before the intervention. In addition, we observed that a 4-week *Chlorella*-derived multicomponent supplementation increased the V̇O_2_max in male university students with insufficient dietary habits, although there was no association between the increase in the V̇O_2_max and circulating levels of vitamin B_2_.

## Figures and Tables

**Table 1:** Anthropometric characteristics

		Pre	Post
Age (years)	Placebo	20.1 ± 0.3	—
	*Chlorella*	19.6 ± 0.3	—
Height (cm)	Placebo	172.8 ± 1.8	—
	*Chlorella*	172.4 ± 1.5	—
Body mass (kg)	Placebo	70.0 ± 3.5	69.6 ± 3.5
	*Chlorella*	69.2 ± 2.7	68.8 ± 2.8
Body fat mass (kg)	Placebo	16.0 ± 1.3	15.3 ± 0.9
	*Chlorella*	14.6 ± 0.9	14.1 ± 0.9

Data are shown as the mean ± SE.

**Table 2:** Nutrient intake and sufficiency rate

Nutrient	Placebo group		*Chlorella* group				
			Baseline			With supplement	
	Mean ± SE	SR (%)	Mean ± SE	SR (%)		Mean ± SE	SR (%)
Energy intake (kcal/d)	2,221 ± 281	82.4	2,055 ± 107	75.9		2,078 ± 107*	76.7
Protein (g/d)	77 ± 13	128.7	64 ± 4	106.6		67 ± 4*	112.3
Fat (g/d)	58 ± 9	—	53 ± 3	—		54 ± 3*	—
n-6 (g/d)	11.3 ± 1.5	102.8	10.6 ± 0.8	96.5		10.7 ± 0.8*	97.3
n-3 (g/d)	2.6 ± 0.3	131.8	2.3 ± 0.3	116.3		2.4 ± 0.3*	121.0
Carbohydrate (g/d)	325 ± 43	—	317 ± 21	—		318 ± 21*	—
Na (mg/d)	5,530 ± 785	921.6	4,444 ± 268	740.6		4,446 ± 268*	740.9
K (mg/d)	2,175 ± 318	87.0	1,976 ± 233	79.0		2,030 ± 233*	81.2
Ca (mg/d)	459 ± 78	57.3	400 ± 40	50.0		418 ± 40*	52.3
Mg (mg/d)	242 ± 36	71.2	202 ± 17	59.4		223 ± 17*	65.5
P (mg/d)	1,048 ± 164	104.8	888 ± 52	88.8		982 ± 52*	98.2
Fe (mg/d)	8.0 ± 1.2	114.7	6.6 ± 0.6	94.5		14.4 ± 0.7*	**205.9**
Zn (mg/d)	9.5 ± 1.4	95.2	8.4 ± 0.5	84.4		8.5 ± 0.5*	85.0
Cu (mg/d)	1.29 ± 0.17	143.7	1.17 ± 0.08	130.2		1.18 ± 0.08*	131.6
Mn (mg/d)	3.4 ± 0.3	85.6	3.2 ± 0.2	79.8		3.2 ± 0.2	79.8
Retinol equivalent (µg/d)	735 ± 123	86.4	586 ± 110	68.9		611 ± 110*	71.9
Vitamin D (µg/d)	10.3 ± 1.6	186.7	8.0 ± 1.4	145.6		26.3 ± 1.5*	**478.7**
Vitamin K (µg/d)	312 ± 55	208.3	287 ± 56	191.4		359 ± 56*	**239.3**
α-Tocopherol (mg/d)	6.8 ± 1.0	105.2	6.3 ± 0.6	97.3		6.5 ± 0.6*	99.6
Vitamin B_1_ (mg/d)	0.8 ± 0.1	57.1	0.7 ± 0.1	51.7		0.8 ± 0.1*	59.1
Vitamin B_2_ (mg/d)	1.2 ± 0.2	75.4	1.1 ± 0.1	66.7		1.4 ± 0.1*	**86.2**
Niacin (mg/d)	16.5 ± 2.7	109.9	13.2 ± 1.2	87.8		16.2 ± 1.3*	**107.8**
Vitamin B_6_ (mg/d)	1.2 ± 0.2	84.5	1.0 ± 0.1	72.2		1.1 ± 0.1*	80.6
Vitamin B_12_ (µg/d)	8.6 ± 1.46	357.0	6.1 ± 0.9	255.2		6.2 ± 0.9*	256.1
Folic acid (µg/d)	305.9 ± 41.3	127.5	264.0 ± 40.3	110.0		264.2 ± 40.3*	110.1
Pantothenic acid (mg/d)	6.9 ± 1.1	137.7	6.1 ± 0.4	122.5		6.4 ± 0.4*	127.9
Vitamin C (mg/d)	89.1 ± 14.7	89.1	88.3 ± 21.1	88.3		88.5 ± 21.1*	88.5

SR, sufficiency rate.*p<0.05 vs baseline. There were no differences in the daily nutrient intake between the groups before the tablet intake period. The SR of iron, vitamin B_2_, D and K, and niacin increased by ≥20% with *Chlorella* supplementation (shown as bold font).

## Abbreviations

AI: adequate intake
ANOVA: analysis of variance
BDHQ: brief, self-administered dietary history questionnaire
EAR: estimated average requirement
HR: heart rate
RDA: recommended dietary amount
RPE: ratings of perceived exertion
V̇O_2_max: maximal oxygen uptake
